# Items and Selections

**Published:** 1872-10

**Authors:** E. N. Brush


Items and Selections.—*By E. N. Brush*.—In a paper read before the Michigan State Medical Society, Prof. Theodore A McGraw gives the history of an Amputation at the Hip Joint. After giving the patient's history to some length and an account of previous operations which the patient had undergone to remove the disease (Osteo-Sarcoma) he proceeds as follows:

I made, therefore, an appointment for the purpose of amputating the thigh in the upper third.

On March 4th, assisted by Drs. Heaton, Foster, Gibbs and Carstens of Detroit, Dr. Carlton of Trenton, Dr. ——— of Dearborn, and numerous medical students, I performed the operation proposed, making large lateral flaps and sawing the bone just below the throchanters. When I had ligated the arteries, and came to examine the bone, I found that its medullary cavity was full of a thick, yellowish matter, not unlike pus, the result, as I believe, of absorption of fluids from the tumor, and consequent degeneration of the medullary membrane and the cancellous tissue. This condition of the bone left no alternative but its entire removal. This was done without great difficulty and with but little further injury to the soft tissues.

The stump was thorougly washed with a solution of carbolic acid, a pledget of lint was inserted into its lower corner to secure free drainage, and the flaps were then brought together with sutures.

It was at a time when Dr. Andrews was experimenting with oxygen gas. He advised its employment in this case as an arterial stimulant. I consented to its use without very much confidence in its powers, chiefly because the stomach of the patient was, for some days, so irritable that it was difficult at times to give stimulants by the mouth. I was inexpressibly gratified by the result. Whenever the patient seemed about to sink under the shock and exhaustion consequent upon the operation, the house physician, Dr. Foster, would have him inhale from half a gallon to a gallon of oxygen, and it was very gratifying to see the almost immediate change for the better. The color would return to the pale face, the pulse would increase in volume, the respiration grow more full and easy, and the nausea temporarily cease.

I cannot, from this experience, too highly recommend the use of this agent in similar cases of shock and exhaustion after severe injuries.

The patient, after the first few days, improved steadily and surely. His strength was kept up by the free use of stimulants and large quantities of gruel, beef-tea, egg-nog, milk-punch, and other nutritious articles of diet. His stump was thoroughly cleansed several times a day with antiseptic injections. About one month after the operation, he was attacked with a superficial form of erysipelas, which was at that time prevailing in Detroit. He recovered, however, without much apparent suffering. Eight weeks after the operation, he began to get about on crutches. Three months after that date, he was driven in a buggy a distance of nearly two miles and assisted up two flights of stairs to get the photographs taken which accompany this paper. * * * * *

'As regards the operation itself, I cannot think that amputation at the hip would be much, if any, more dangerous than that immediately below the trochanters, if performed with the same incisions through the soft parts. It is my opinion that the operative procedures usually adopted increase the unavoidable risk by sacrificing an unnecessary amount of soft tissues. Flaps which will amply cover the stump of an amputation below the trochanter are not a bit too large for a disarticulation at the hip joint. The resulting cushion is better adopted to support pressure, and the immediate shock of the operation is much less than when the flaps are made to accurately fit the exposed surfaces. That the disarticulation is easily performed, with the reservation of so much tissue, was proven by the case whose history I have just given.

A disarticulation thus performed is, in some respects, preferable to a high amputation through the continuity. The patient escapes all risk of osteo-myeletis, and, in cases of malignant disease, has a vastly better chance of escaping its recurrence.

The *British Medical Journal* reports a death from chloroform in the Great Nortnern Hospital. The patient was a man 53 years old and was about to undergo the operation of Lithotomy.——The authorities of Chicago have taken a step which will meet with hearty commendation in prohibiting the posting of hand bills of quacks and quack medicines about the streets.

Dr. J. H. B. McClellan, reports *American Journal of Medical Sciences*, the extirpation of a fibrord tumor necessitating the removal of the entire parotid gland, ligation of the external carotid artery and jugular vein, and division of the Portio Dura Nerve, the case terminated favorably and at the end of eleven months the disease manifests no tendency to return.——In the *American Journa' of Medical Sciences* for Oct., Dr. S. T. Knight reports the successful removal of an ovarian tumor by enucleation. The Doctor was unwilling to trust the pedicle to itself, but at the conclusion of the operation applied a clamp.——In a report of forty-eight cases of subperiostial resection of the elbow (*London Lancet*) M. Ollier says that, with proper care and skilfulness in dissecting the entire periostial sheath, the bones are not only reproduced in their continuity, but with their heads and articular tuberosities, so as to permit the play of the joint in all its movements. It is necessary to employ minute precautions in the after-treatment for obtaining the above results. Slight movements must be made at an early time with the joints. Anchylosis is not to be dreaded but rather too great mobility of the joint, especially in individuals from forty to fifty years of age.——The *British Medical Journal* for July 20th, contains an account by Mr. H. Grace of a remarkable case of injury of the spine. A man while at work in a coal mine was crushed by a heavy stone falling upon him. He was so doubled up that his head rested between his thighs and his perinæum upon his heels. When first seen by a medical man he was cold, breathing hurriedly and evidently sinking.

The first, second, third and fourth lumbar vertebræ all appeared to be implicated in the injury. He was placed on a mattress and by the aid of four men, two at each end, extension made, when the spinal column resumed its normal position with an audible snap. The breathing and pulse were immediately relieved. For two months it was necessary to pass the catheter daily, after which the urine escaped involuntarily, and has continued to do so up to the present time.

At the end of two and a half years he was able to resume his work as a collier. "The sensibility of the soles of feet, and in the posterior portion of both legs, is much impaired; and around the arms perinæum inner part of both buttocks, scrotum and penis it is *entirely* destroyed." At the time of the accident he was married and has had two children born to him since. Mr. Grace draws some valuable observations concerning the functions of certain nerves from this case.

Dr. H. Knapp reports in the *Medical Record*, forty-one cases of blindness or deafness resulting from Cerebro-Spinal Meningitis, which have come under his care.

